# Research on spatial and temporal differences of carbon emissions and influencing factors in eight economic regions of China based on LMDI model

**DOI:** 10.1038/s41598-023-35181-w

**Published:** 2023-05-17

**Authors:** Pan Jiang, Xiujuan Gong, Yirui Yang, Kai Tang, Yuting Zhao, Shu Liu, Liang Liu

**Affiliations:** 1 School of Economics and Management, Southwest University of Science and Technology, Mianyang, 621010 China; 2 School of Environment and Resource, Southwest University of Science and Technology, Mianyang, 621010 China; 3 School of Science, Southwest University of Science and Technology, Mianyang, 621010 China

**Keywords:** Sustainability, Environmental economics

## Abstract

With the gradual increase of international willingness to reach the carbon peak and carbon neutrality, this paper decomposes the influencing factors of China’s carbon emission changes from 2008 to 2019 using the Logarithmic Mean Divisia Index model (LMDI), and analyzes the contribution amount and rate of each influencing factor. The results found that: for the whole country, the cumulative growth of carbon emissions during the study period is about 416,484.47 (10^4^ tons), among which the economic growth effect plays a major role in promoting, with a cumulative contribution rate of 284.16%; The increase in regulation intensity and the optimization of industrial structure, however, suppress carbon emissions well, with a cumulative contribution rate of about – 199.21% and − 64.75%, respectively, during the study period. For economic regions, the cumulative influence direction of each driver is the same as that of the whole country, while the population size effect in the northeast economic region and the regulation input effect in the eastern coastal economic region act in the opposite direction from other economic regions, and the carbon emission reduction direction of the energy intensity effect varies from one economic region to another. Accordingly, this paper proposes policy recommendations to enhance regulatory intensity, optimize industrial and energy consumption structure, implement localized emission reduction strategies, and promote synergistic emission reduction in economic zones.

## Introduction

The climate issue brought about by carbon emissions has become a global concern. According to the report of the United Nations Office for Disaster Risk Reduction^1^, global warming increases the temperature by 1 °C, and the incidence of daily extreme precipitation events may increase by 7%. From 2000 to 2019, extreme weather caused by global warming has killed more than 500,000 people, affected 390 million people, and caused economic losses of 2.97 trillion US dollars^2^. China has become the biggest player in the global carbon reduction field, with the world’s largest energy production, consumption and energy imports. China's economic development and carbon emission trends have gradually become the focus of global attention^3^. At the 75th session of the UN General Assembly in 2020, China proposed to strive for peaking carbon emissions by 2030 and carbon neutrality by 2060. It is not only a major decision made by China based on the overall situation of the country’s development, but also a major measure to cope with complex environmental threats and gain strategic advantages for the country.

At present, China is still in the middle to late stages of industrialization, and under the background of the new economic normal, the task of reducing carbon emissions is still very arduous^4–6^. There are differences in the economic structure, resource endowment, and institutional environment of each region^7^, and carbon emissions also have different characteristics^8,9^. Thus, policy implementation should not only emphasize the regional differences in the paths of carbon peaking and carbon neutrality but also avoid “one size fits all” and phenomena that do not conform to local actual conditions. Therefore, it is important for regional and national ecological environment governance to clarify the factors affecting China’s carbon emissions, especially the targeted analysis of economic zones.

With China's increasing willingness to achieve a carbon peak by 2030, scholars at home and abroad have conducted a lot of research on China’s carbon emissions, focusing on the feasibility extrapolation and realization path of China's 3060 plan, carbon emission efficiency analysis^10^, carbon emission peak projection^11^, carbon emission impact factor decomposition^12,13^, carbon emission research and emission reduction strategies by industry and region^13^. For carbon emissions research, scholars have mainly used the DEA model, IPAT model, Kaya constant equation, LMDI decomposition method, environmental Kuznets curve, etc.^14^. Compared with the extended Kaya constant equation, the LMDI decomposition model can decompose each influence factor into data without residual terms^15^, which has the advantages of strong interpretability, unique results, easy-to-use and mature technology, and is often applied to carbon The relevant studies have revealed that the factors influencing the level of carbon emissions in China are mainly economic growth level, energy consumption, technological progress, urbanization rate, and industrial structure^16,17^. However, little research has been done on the impact of environmental regulation, which is an institutional arrangement that uses new concepts, systems and institutions to restrain the behavior of the public to conserve resources and protect the environment, and plays a very important role in the process of environmental governance^18^. Therefore, this paper also uses the LMDI model as a basis when mining the factors influencing carbon emissions, and innovatively introduces environmental regulation indicators for driving analysis. To remedy the shortcomings of previous studies, we aim to identify carbon emission drivers more scientifically and precisely, and to provide a scientific basis for the formulation of sensible badlands policies to reduce CO_2_ emissions and promote low-carbon green development.

This study also fills a research gap by extending environmental regulation to a decomposition analysis of the main drivers of CO_2_ emissions in terms of China and economic regions. The rest of the study is organized as follows: Chapter 2 literature review, a brief compilation, and analysis method. Chapter 3 describes the methodology and data sources. Chapter 4 provides an analysis and discussion of the results; Chapter 5 concludes and policy recommendations.

## Literature review

In the 1990s, Grossman and Panayotou proposed the Environmental Kuznets Curve (EKC)^19,20^. The theory suggests that in the early stages of economic development, the level of environmental pollution increases with the growth of per capita income, and when economic growth exceeds a critical level, environmental pollution decreases, i.e. there is an inverted “U” shaped relationship between economic growth and environmental pollution^21^. Since then, scholars have argued for the EKC in terms of national policies^22^, value chains^23^, and participating actors^24^. However, most of these studies treat the relationship between economic growth and environmental pollution as a “black box”^25^, and only use economic output data and environmental data to explore the degree of mutual influence between the two, without explaining the specific mechanisms of influence between specific economic factors such as industrial structure and technology level and environmental factors such as resource inputs and environmental pollution emissions. The mechanism of interaction between specific economic factors such as industrial structure and technology level and environmental factors such as resource inputs and environmental pollution emissions is not explained. However, it is more important to dissect the “black box” to explore the mechanisms behind the EKC^26^. In this context, factor decomposition models have been introduced into the study of the EKC. Due to their ability to break down indicators into components and assess the contribution of each component^27^, factor decomposition models are widely used to explore the mechanisms underlying environmental issues such as energy consumption and carbon emissions^28^.

There are two main types of common carbon emission factor decomposition models, Structural Decomposition Analysis (SDA) and Index Decomposition Analysis (IDA). Since researchers first used it to analyze industrial electricity consumption in the early 1980s, Index Decomposition Analysis (IDA) has been widely used in energy and emissions studies^29^. In 2001, the LMDI-I (Additive Model) and LMDI-II (Multiplication model) were formally introduced^30^. Logarithmic Mean Divisia Index (LMDI) has become the most popular IDA method because of its residual-free, complete decomposition, ease of operation, and applicability^31^. The Japanese scholar Yoichi Kaya, who linked CO_2_ emissions to economic, energy and demographic factors, proposed the Kaya identity to examine the degree of influence of each driver on CO_2_ emissions^32,33^. Since then, scholars have continued to expand on the Kaya-LMDI. Xue Wang included the industrial structure and investment efficiency in the Kaya-LMDI^34^, while Xiaojun Ma expanded the Kaya-LMDI to include six drivers: carbon emission factor, energy structure, energy intensity, industrial structure, economic output and population size^35^. In recent years, Python-LMDI developed based on Python tools has appeared, which simplifies the calculation process of LMDI, and extends the range of applications of LMDI with its advantages of convenience, speed, and efficiency^36^. Table 1 lists the research results for the last 5 years. Current research trends indicate that LMDI is expected to continue to play an important role in future research on our carbon emissions.

**Table 1:** Literature on the decomposition of China’s regional carbon emission factors in the past 5 years.

Author	Research period	Basic model	Research region
XiaoyuLi^37^	2012–2017	SDA	South & North
Shichun Xu^38^	2002–2012	SDA	Jiangsu province
Jingli Fan^39^	1997–2013	SDA	Beijing-Tianjin-Hebei region
Feng Chen^12^	2003–2019	LMDI	Yellow river basin
Xue Wang^34^	2002–2017	LMDI	Northeast region
Shaohua Yang^40^	2000–2019	LMDI	Yangtze river economic belt
YaoZhang^41^	2000–2020	LMDI	Xi’an city
YingYu^42^	2006–2019	LMDI、SDA	Pearl river delta
Quande Qin^43^	2004–2017	LMDI	30 provinces
XuYang^44^	1995–2018	LMDI	Chang-Zhu-Tan urban agglomeration

As a vast country, China faces significant differences in terms of energy endowments, natural conditions and regional economic development^45^. Therefore, it is necessary to differentiate the analysis according to the regional characteristics. Throughout the existing regional studies of China’s carbon emissions, there is a large body of literature on on the division of many regions according to the geographical location in the east-central-west region or the traditional seven geographic regions^46^. While this classification is easy to apply, it cannot be directly used to find targeted strategies, as the same measures do not necessarily apply to provinces in close geographical proximity^47^. And, as China’s economy enters a phase of high-quality development, the internal differences within the traditional regional analysis are coming to the fore. Literature is emerging that uses urban agglomerations^14,48^, river basins^12,49^, etc. as a divisional perspective. To further broaden the scale of regional studies, some scholars have divided the 30 provinces into eight clusters based on the *Strategies and Policies for Coordinated Regional Development* published by the Development Research Center of the China State Council, to identify reasonable industrial restructuring directions and “carbon peak and carbon neutrality goals” action plans for each region according to the characteristics of carbon emissions in different regions^50^.

In summary, scholars have conducted in-depth studies on the factors that influence China's regional carbon emissions, but it is rare to see environmental regulation factors included in the LMDI decomposition models when decomposing carbon emission factors; and there is also little literature on comparative analysis of China’s eight economic regions when analyzing regional carbon emissions. Therefore, based on the existing studies, this paper uses data from 30 Chinese provinces and cities from 2008 to 2019, establishes an LMDI-I additive decomposition model based on the Kaya identity, analyses the factors influencing carbon emissions including environmental regulation factors, explores the spatial and temporal differences in the factors influencing carbon emissions between the whole country and the eight economic regions, and proposes a reasonable evolutionary path for low-carbon development based on the LMDI decomposition results, so as to provide lessons for the low-carbon development of other regions. The analysis will also provide a reasonable path for low-carbon development and lessons for other regions to learn.

## Data and methods

### Data source

This paper takes the eight comprehensive economic zones delineated in the report “*Strategies and Policies for Coordinated Regional Development*” by the Development Research Center of the State Council as the research object and conducts a study on carbon emissions in China as a whole and in the eight economic zones based on relevant data from 2008 to 2019 (Tibet, Hong Kong, Macao, and Taiwan are not included in the sample set due to missing data), and the specific regional scope is shown in Table 2.

**Table 2:** Range of eight economic zones in China.

Name	Code	Regional scope
Northeast economic zone	NEEZ	Liaoning, Jilin, Heilongjiang
Northern coastal economic zone	NEZ	Beijing, Tianjin, Hebei, Shandong
Eastern coastal economic zone	ECEZ	Shanghai, Zhejiang, Jiangsu
Southern coastal economic zone	SCEZ	Fujian, Guangdong, Hainan
Mid-Yellow river economic zone	MYeREZ	Shanxi, Inner Mongolia, Henan, Shaanxi
Mid-Yangtze river economic zone	MYtREZ	Hubei, Hunan, Jiangxi, Anhui
Southwest economic zone	SWEZ	Yunnan, Guizhou, Sichuan, Chongqing, Guangxi
Northwest economic zone	NWEZ	Gansu, Qinghai, Ningxia, Xinjiang

The data for measurement analysis were mainly obtained from IPCC(2006), CSMAR, *China Statistical Yearbook*, *China Energy Statistical Yearbook*, *China Science and Technology Statistical Yearbook*, and the statistical yearbooks of each province from 2008 to 2019, and the descriptive statistics of each variable are shown in Table 3.

**Table 3:** Descriptive statistics of the sample data.

Indicator name	Code	Unit	Data source
Total carbon emissions	C	10^4^ tons	CSMAR, IPCC(2006)
Energy consumption	E	10^4^ tons of standard coal	CSMAR, China energy statistical yearbook
Industrial pollution control completed investment amount	I	10^4^ yuan	China statistical yearbook
Value added of secondary industry	V	Billion	China statistical yearbook
Gross regional product	GDP	Billion	China statistical yearbook
Year-end resident population	P	10^4^ people	China statistical yearbook

The total energy consumption is calculated according to the primary energy statistics and the standard coal conversion factor. Data from CSMAR, so the detailed calculation steps are not listed.

### Model building

In this paper, we decompose the factors influencing CO_2_ emissions based on the modified equation of Kaya’s^32,51^ identity as: Energy Carbon Emission Intensity effect (ECEI), Regulatory input effect(RIE), Regulatory strength effect(RSE), Industry Structure Effect(ISE), Economic growth effect(EGE) and Population size effect(PSE)^11,52^, show as Eqs. (1) and (2):

1 $$\left\{\begin{array}{c}C={\sum }_{i}{C}_{i}=\frac{C}{E}\times \frac{E}{I}\times \frac{I}{V}\times \frac{V}{GPD}\times \frac{GDP}{P}\times P\\ ECEI=\frac{C}{E}\\ RIE=\frac{E}{I}\\ RSE=\frac{I}{V}\\ ISE=\frac{V}{GDP}\\ EGE=\frac{GDP}{P}\\ PSE=P\end{array},\right.$$

where, C is the total CO_2_ emissions, data from CSMAR, refer to IPCC (2006). E is the total energy consumption, I is the amount of investment completed in industrial pollution control for the year, V is the value added of secondary industry, GDP is the gross regional product for the year, and P is the year-end resident population.

In summary, Eq. (2) can be derived:

2 $$C={\sum }_{i}ECEI\times RIE\times RSE\times ISE\times EGE\times PSE.$$

Additive and decomposition theory based on LMDI model^29^, drawing on the Python-LMDI model developed by Xiang^36^ et al. decompose the CO_2_ emission growth into ΔECEI, ΔRIE, ΔRSE, ΔISE, ΔEGE and ΔPSE, then the change in CO_2_ emissions in year T relative to the base year is expressed as Eq. (3):

3 $$\Delta C={C}_{T}-{C}_{0}=\Delta ECEI+\Delta RIE+\Delta RSE+\Delta ISE+\Delta EGE+\Delta PSE$$

The contribution of each factor change to the change in CO_2_ emissions is calculated as Eq. (4):

4 $$\left\{\begin{array}{c}\Delta ECEI={\sum }_{i}\frac{{C}_{T}-{C}_{0}}{\mathrm{ln}{C}_{T}-\mathrm{ln}{C}_{0}}\mathrm{ln}\left(\frac{{ECEI}_{T}}{{ECEI}_{0}}\right)\\ \Delta RIE={\sum }_{i}\frac{{C}_{T}-{C}_{0}}{\mathrm{ln}{C}_{T}-\mathrm{ln}{C}_{0}}\mathrm{ln}\left(\frac{{RIE}_{T}}{{RIE}_{0}}\right)\\ \Delta RSE={\sum }_{i}\frac{{C}_{T}-{C}_{0}}{\mathrm{ln}{C}_{T}-\mathrm{ln}{C}_{0}}\mathrm{ln}\left(\frac{{RSE}_{T}}{{RSE}_{0}}\right)\\ \Delta ISE={\sum }_{i}\frac{{C}_{T}-{C}_{0}}{\mathrm{ln}{C}_{T}-\mathrm{ln}{C}_{0}}\mathrm{ln}\left(\frac{{ISE}_{T}}{{ISE}_{0}}\right)\\ \Delta EGE={\sum }_{i}\frac{{C}_{T}-{C}_{0}}{\mathrm{ln}{C}_{T}-\mathrm{ln}{C}_{0}}\mathrm{ln}\left(\frac{{EGE}_{T}}{{EGE}_{0}}\right)\\ \Delta PSE={\sum }_{i}\frac{{C}_{T}-{C}_{0}}{\mathrm{ln}{C}_{T}-\mathrm{ln}{C}_{0}}\mathrm{ln}\left(\frac{{PSE}_{T}}{{PSE}_{0}}\right)\end{array}.\right.$$

The contribution of changes in each factor to the change in CO_2_ emissions is expressed as Eq. (5):

5 $${D}_{X}=\frac{\Delta X}{\Delta C}\times 100\%.$$

X represents different drivers of CO_2_ emissions.

## Results analysis

### National level analysis

**Figure 1:**
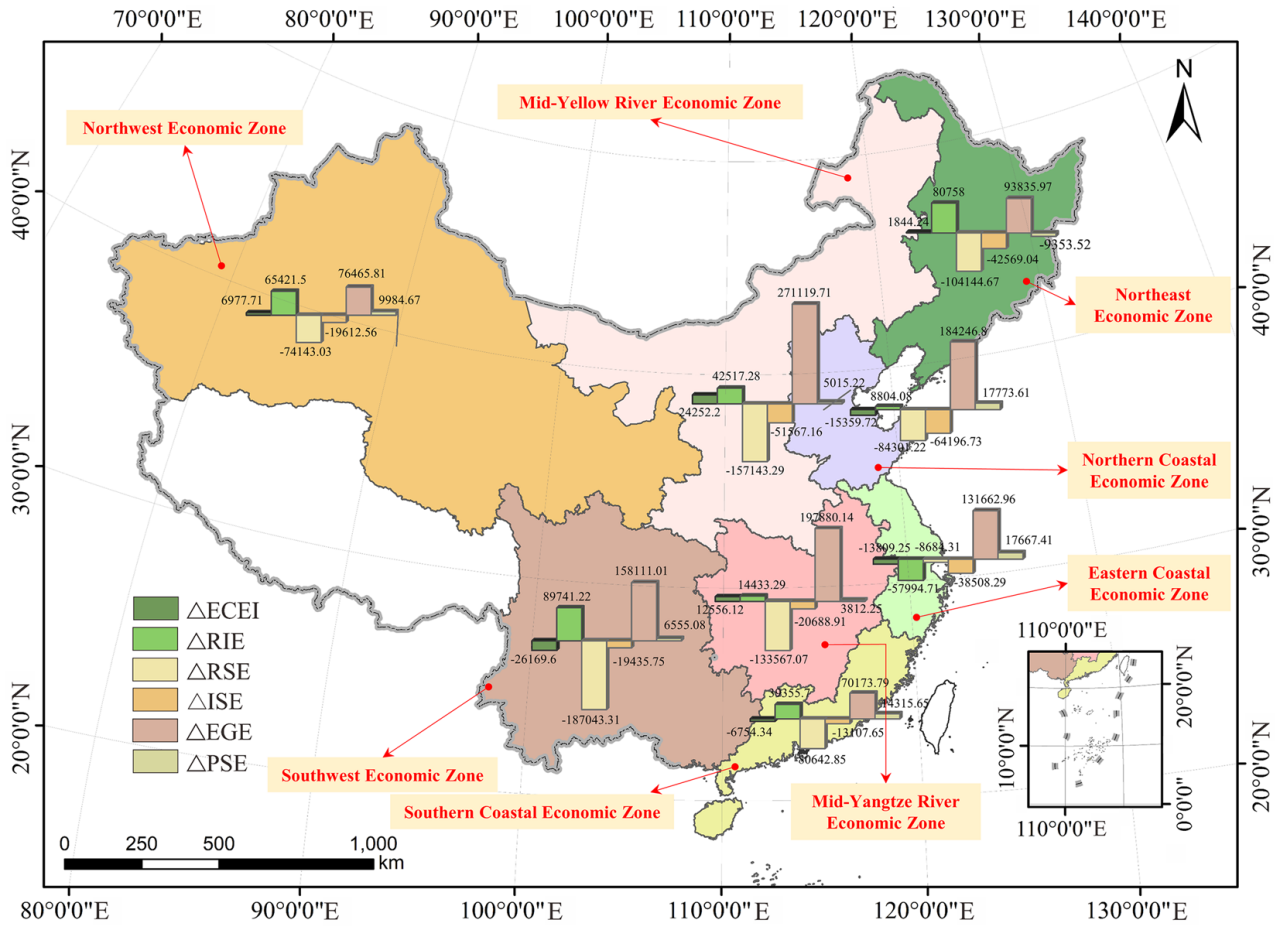
Cumulative effect of each influencing factor from 2008 to 2019. (No. GS(2020)4632, No data in the white area).

Based on the time series data of China from 2008 to 2019, the effects of energy intensity, regulatory inputs, regulatory intensity, industrial structure, economic growth, and population size on carbon emissions in China are empirically analyzed by combining the decomposition results of the Python-LMDI model. The combined effect of the influencing factors over the study period is shown in Fig. 1 (Drawn through ArcGIS 10.7: https://www.esri.com/zh-cn/arcgis/products/arcgis-desktop/overview and PowerPoint 365: https://www.microsoft.com/zh-cn/microsoft-365/powerpoint).

For the years 2008 to 2019, regulation intensity and regulation input have the greatest impact on carbon emission change, but in opposite directions, with regulation intensity effect being the greatest inhibitor of carbon emission growth, contributing about − 191.69% of the cumulative emissions reduction from 2008 to 2019. Except for 2015, the impact curve of the environmental regulation input effect on carbon emission change generally shows an inverted "U" shape, with the critical value point or "inflection point" in 2013. On the contrary, except for 2015, the impact curve of environmental regulation intensity effect on carbon emission change has an overall "U" shape, but the critical point or "inflection point" is also in 2013. The possible reasons for this are that in February 2013, the Chinese Ministry of Environmental Protection issued the *"12th Five-Year Plan for the Development of National Environmental Protection Standards"*, which revised environmental standards and increased environmental investment; in September of the same year, the State Council issued the *"Notice on the Action Plan for the Prevention and Control of Air Pollution"*, referred to as the "Ten Measures for Air Pollution". In September of the same year, the State Council issued the Circular on the Action Plan for the Prevention and Control of Atmospheric Pollution, referred to as the "Ten Atmospheric Articles", which set new goals for air pollution prevention and control by proposing many specific measures to increase comprehensive treatment and reduce emissions of multiple pollutants. The introduction of two consecutive environmental regulation policies has raised awareness and increased investment in environmental protection in all regions of the country, so that the contribution of the environmental protection input effect to carbon emissions reached a maximum value of – 80,339.23.

**Figure 2:**
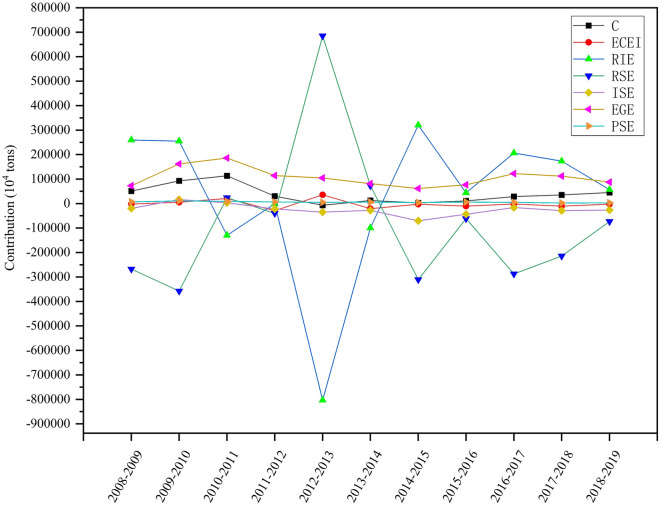
Cumulative contribution of carbon emission driving effects in China 2008–2019.

As an inverse indicator, the contribution of the environmental regulation input effect decreases rapidly and the contribution of the environmental regulation intensity effect increases to a maximum value of 685,203.82. 2015, the closing year of the 12th Five-Year Plan, is the year in which the new plan will begin, which will change the original situation. This may be the reason for the outliers in 2015 (Fig. 2).

The level of economic growth is the third most important factor influencing changes in carbon emissions. As the level of the economy improves, GDP per capita increases, and industry and urbanization continue to grow, the demand for carbon emissions will also increase, so economic growth is a positive effect. The contribution value of the economic growth level effect keeps increasing between 2009 and 2011 and then decreases, presumably because since the 2008 economic crisis, there has been an increase in infrastructure construction in places to accelerate the economic recovery, which promotes the growth of carbon emissions. After that, the government continued to increase environmental protection and put the environment and economic development side by side, and the incremental carbon emissions caused by economic growth decreased. The fourth major factor driving changes in carbon emissions is industrial structure. The contribution of industrial structure is negative all year round, although, in 2010 and 2011, the contribution becomes positive, 16,380.02 (10^4^ tons) and 3271.06 (10^4^ tons) respectively, in general, the effect of industrial structure limits the increase of carbon emissions. This also reflects that the third technological revolution, represented by information technology and new materials technologies, has impacted traditional industries, and China's industrial structure, which is dominated by secondary industries, is changing.

As a negative indicator, the contribution of energy intensity fluctuated significantly between 2010–2011 and 2012–2013, first from positive to negative and then from negative to positive. The reason for this fluctuation may be that during the 12th Five-Year Plan period, China's energy structure has been optimized, clean energy has been developed rapidly, and the competition and substitution between clean energy represented by hydro, wind, and solar energy and traditional energy represented by coal have become increasingly intense, and the traditional energy structure has been adjusted, thus the contribution of energy intensity effect is unstable. The driving effect of population size on changes in carbon emission has been stable over time and has been small during 2008 to 2019 period, with a contribution rate ranging from 6 to 80% over time, and is the factor with the least impact on carbon emissions. The likely cause is the long-standing family planning policy and the sense of birth control people have developed. Although the population expansion policy was promulgated in 2015, the liberalization of the fertility policy did not trigger a fertility climax, and China’s fertility level and the birth population did not get the imagined increase, and China’s population did not get a large-scale expansion but tended to be stable in the long term, and it is reasonable to believe that the population size will not increase significantly in the next few years, so the population size has the smallest and most stable impact on carbon emissions.

### Regional level analysis

#### Regional carbon emissions patterns

Table 4 shows the cumulative effect of carbon emission changes in the eight economic regions of China from 2008 to 2019. In terms of the amount of carbon dioxide change, the Northeast Comprehensive Economic Zone had the least growth in carbon emissions during the study period, with an increase of 20,370.98 (10^4^ tons). The overall performance is MYeREZ > MYtREZ > NWEZ > NCEZ > ECEZ > SCEZ > SWEZ > NEEZ. In terms of the magnitude of change, NWEZ had the largest increase in carbon emissions of 146.7% from the study base period. MYeREZ and MYtREZ growth followed; the SCEZ has the middle of the pack in terms of carbon emissions growth (40.52%). While the NCEZ and ECEZ coasts, which are also coastal economic zones, are basically the same, with a growth rate of about 26%. NEEZ was the lowest, with only a 20.72% increase from the study base period. The magnitude of the change is shown overall: NWEZ > MYtREZ > MYeREZ > SCEZ > ECEZ≈NCEZ > SWEZ > NEEZ (Table 4).

**Table 4:** Cumulative effects of carbon emission changes in eight economic zones.

Area	CO_2_ volume in 2008 (10^4^ t)	Change from the base period (10^4^ t)	Rate of change over the base period
NEEZ	98,339.2197	20,370.98	20.72%
NCEZ	177,913.8238	46,966.84	26.40%
ECEZ	114,054.0298	30,333.83	26.60%
SCEZ	57,598.62103	23,340.3	40.52%
MYeREZ	209,972.7599	134,193.96	63.91%
MYtREZ	114,947.7613	74,425.82	64.75%
SWEZ	101,760.7497	21,758.63	21.38%
NWEZ	44,371.49658	65,094.11	146.70%

In summary, total carbon dioxide emissions are increasing in all eight economic regions, and the situation for carbon reduction remains relatively severe. The NEEZ is the smallest in both total volume and magnitude. This is because the NEEZ, as a heavy equipment and equipment manufacturing base, has benefited from the rapid transformation of its industrial structure in recent years and has played a very significant role in carbon reduction. NWEZ, MYtREZ and MYeREZ are in the top three in terms of volume and rate of change. Among them, the two economic zones in the middle reaches of the MYeREZ and the MYtREZ, as China's main grain base and heavy industrial production base, both have the difficulties of low openness to the outside world and the difficult task of industrial restructuring, which in turn leads to an increase in carbon emissions. The NWEZ ranks behind all regions in terms of economic size as a percentage of the country and the NWEZ is also the largest region in China and the fastest growing region in terms of economic growth. It is a key breakthrough direction for the future to use the Belt and Road strategy and modern industrial means to transform the ecological environment in northwest China so that the population can grow rapidly and bring about rapid economic growth.

#### Regional differentiation analysis

In terms of influencing factors, for all integrated economic zones, the level of economic growth is an important indicator of the contribution to the increase in carbon emissions, with a contribution rate above 100%. Slightly different from the findings of Ding et al.^50^ is that the extended analysis of the impact factor indicators reveals that the effect of economic growth level is most prominent in the southwest, with a contribution rate of 726.66%. At the same time, the clean energy resource of the SWEZ endowment and policy support enable it to grow at a high rate while maintaining low incremental carbon emissions due to interactions. The NWEZ, which is also a concentration of energy resources in China, has the smallest contribution of economic growth level to carbon emissions (117.47%). Because most of the provinces in the NWEZ are in the process of moving from agriculture to industrialization, the level of industrialization and urbanization development is low and the growth rate of the economy is slow compared to the rest of the economic zone^53^.

Human life needs to consume energy, and the energy consumption is an important source of carbon dioxide emissions, and more population means more carbon dioxide emissions, and studies have shown that there is a positive relationship between world population size and carbon emissions^54^; however, on the contrary, the population size effect, except for the Northeast Economic Zone (– 45.92%), it can positively contribute to carbon emissions in other economic zones, which may be related to the Northeast Economic Zone with a single industrial structure and weak manufacturing industry, leading to a large labor force loss and negative population size growth^55^, which is consistent with the results shown in the *2019 National Economic and Social Development Statistical Bulletin* of Liaoning, Jilin, and Heilongjiang provinces.

The results show that the regulatory input effect can promote the increase of carbon emissions in all economic zones (except the ECEZ), with the largest contribution to the SWEZ (412.44%) and the smallest contribution to the NCEZ (18.75%); the ECEZ, on the other hand, because of the early start of modernization and high intensity of R&D investment, has played a significant role in carbon emissions in recent years with the development of scientific and technological innovation, industrial integration and industrial carbon emission efficiency The continuous improvement of carbon emission has played an obvious inhibiting effect on carbon emission, which is consistent with the findings of Mi et al.^56^.

Among the factors that inhibit the increase of carbon emissions, the changes in the regulation intensity effect and the industrial structure effect during the study period are able to inhibit all the integrated economic zones. As an inverse indicator of the regulatory input, the carbon reduction effect of the regulatory intensity effect in the SWEZ is the largest (− 859.63%), which is 30 times larger than that of the smallest ECEZ (− 28.63%), while the carbon reduction effect of the rest of the economic zones ranges from − 113.90% (NWEZ) to − 511.24% (NEEZ). In terms of industrial structure effects, the NEEZ is the most inhibited (− 208.97%), the two economic zones with the weakest inhibitory effect on carbon emission increase are the MYtREZ (− 27.80%) and the NWEZ (− 30.13%). Overall, stronger regulation and restructuring of industry remain the main ways to reduce carbon emissions. In comparison, the implementation of environmental regulation also indirectly promotes the adjustment of regional industrial structure^57,58^, which in turn suppresses carbon emissions, so the role of environmental regulation is stronger than adjusting industrial structure.

The direction of the energy intensity effect on carbon emission reduction is not consistent across economic regions. There is an inhibitory effect on the SWEZ, NCEZ, ECEZ, and SCEZ, with the strongest inhibitory effect in the SWEZ(-120.27%), which is due to the large proportion of clean energy in the SWEZ as the inland energy base^59^, especially the full utilization of hydro energy and natural gas, which significantly promotes regional carbon emission reduction. In contrast, the energy intensity effect promotes carbon emissions in the remaining four economic regions, but to an overall weaker extent, with the middle reaches of the MYeREZ receiving the strongest promotion effect (18.07%) and the middle reaches of the MYtREZ receiving the weakest promotion effect (9.05%); although China vigorously implements energy conservation and emission reduction policies, the low level of low-carbon technology innovation in this regions^60^, the weak prior foundation, and the slow transformation of energy structures have led to energy intensity a certain rebound occurred (Table 5).

**Table 5:** Cumulative contribution of carbon emission sub-influencing factors in eight economic regions of China.

Area	D_C_	D_ECEI_	D_RIE_	D_RSE_	D_ISE_	D_ETE_	D_PSE_
NEEZ	100%	9.05%	396.44%	− 511.24%	− 208.97%	460.64%	− 45.92%
NCEZ	100%	− 32.70%	18.75%	− 179.49%	− 136.69%	392.29%	37.84%
ECEZ	100%	− 45.52%	− 191.19%	− 28.63%	− 126.95%	434.05%	58.24%
SCEZ	100%	− 28.94%	168.62%	− 345.51%	− 56.16%	300.66%	61.33%
MYeREZ	100%	18.07%	31.68%	− 117.10%	− 38.43%	202.04%	3.74%
MYtREZ	100%	16.87%	19.39%	− 179.46%	− 27.80%	265.88%	5.12%
SWEZ	100%	− 120.27%	412.44%	− 859.63%	− 89.32%	726.66%	30.13%
NWEZ	100%	10.72%	100.50%	− 113.90%	− 30.13%	117.47%	15.34%

**Figure 3:**
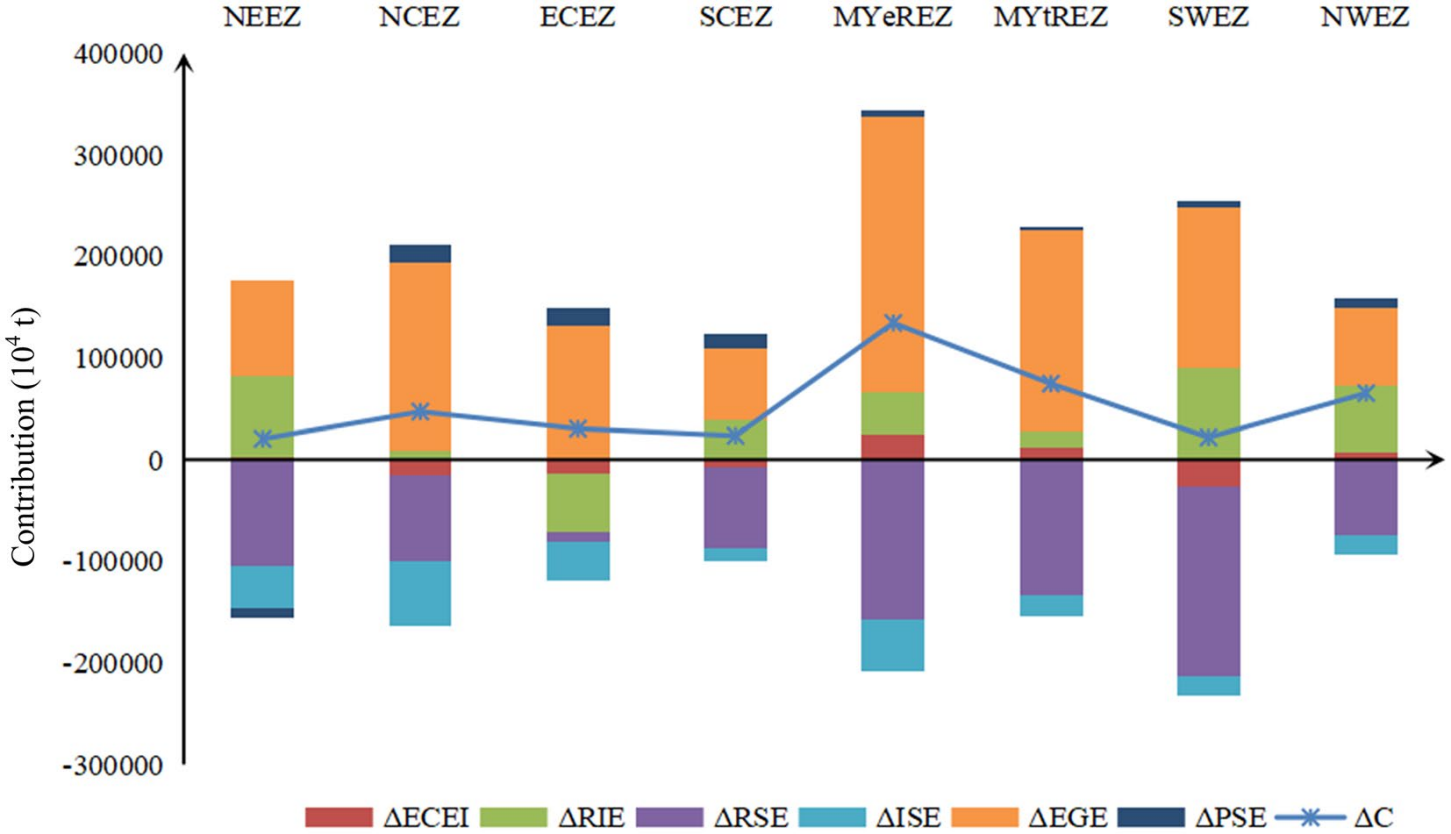
Cumulative contribution of carbon emission sub-influencing factors in eight economic regions of China.

Overall, there are differences in the effects of various factors on carbon emissions in different economic zones, especially after the introduction of environmental regulation factors, which significantly change the results compared to previous studies. For different economic zones, the economic growth level effect is the most important factor that inhibits carbon emission reduction, and the regulatory input effect also plays a strong inhibiting role with the increasing energy consumption (except for the ECEZ); the industrial structure effects and the regulatory intensity effects can significantly promote carbon emission reduction; the population size effect and the energy intensity effect have relatively weak effects on carbon emission reduction, and the energy intensity effect has obvious regional The effects of population size and energy intensity on carbon emission reduction are relatively weak, and the energy intensity effect has obvious regional differences (Fig. 3).

## Conclusions and recommendations

### Conclusions

Based on China's provincial statistics from 2008 to 2019, this paper presents a comprehensive analysis of the spatial and temporal evolution and drivers of carbon emissions at the level of the whole country and the eight integrated economic zones, with the following main findings.

Among the six drivers at the national level, the regulatory input effect and the regulatory intensity effect are the main factors affecting the change in carbon emissions. The curves for both effects show an “inflection point” in 2013 and an abnormal change in 2015, which is slightly related to the promulgation of *the National Environmental Protection Standards 12th Five-Year Plan* and *the Notice of Action Plan for Air Pollution Prevention and Control in 2013*. The policy direction has increased the importance attached to the environment, raised awareness and increased investment in environmental protection in all regions of the country. The reason for the outlier in 2015 may be that it is the closing year of the *12th Five-Year Plan*. Meanwhile, the economic growth effect has been declining since the financial crisis. But with the third technological revolution and the development of clean energy industries, the industrial and energy structures have been continuously adjusted and optimized, and the potential for energy saving and emission reduction from the industrial structures and energy carbon emission intensity effect has been increasing. The population size effect, as a negative carbon emission reduction effect, is stable over the long term and has the smallest driving effect, which is closely related to the stable rate of population growth in China.

At the economic zone level, the Mid-Yellow River Economic Zone had the largest cumulative increase in carbon emissions during the study period, and the Northwest Economic Zone far exceeds the other economic zones in terms of the magnitude of the change. The Northeast Economic Zone, on the other hand, came bottom in both the amount of carbon emissions growth and the magnitude of the change. For all integrated economic zones, the economic growth effect is the main contributor to the increase in carbon emissions, but there is some regional variation in the contribution to the change in carbon emissions. Cumulatively over the study period, the level of economic growth effect is most pronounced in the Southwest and lowest in the Northwest Economic Zone. The regulatory input effect contributes to the increase in carbon emissions in all economic zones (except the Eastern Coastal Economic Zone), with the largest contribution to the Southwestern and the smallest contribution to the Southwestern Coastal Economic Zone. The population size effect positively contributes to carbon emissions in all economic regions except the Northeast Economic Zone. Among the factors that suppress the increase in carbon emissions, regulatory strength effects and changes in industrial structure effects over the study period inhibit all integrated economic regions. However, the energy carbon emission intensity effect is not consistent across economic regions, with the strongest inhibitory effect in the Southwest and the strongest promoting effect in the middle reaches of the Mid-Yellow River Economic Zone.

### Recommendations

Based on the above findings, this paper further proposes the following targeted carbon reduction policy recommendations:

1. The effect of economic growth is the main factor that promotes the increase of regional carbon emissions. Therefore, it is necessary to change the mode of economic growth, pay attention to the impact of population migration and labor loss, pay attention to the cultivation of talents and the research and development of new technologies, support the development of a digital economy, and develop high-tech industry, vigorously develop a low-carbon economy.
2. Pay attention to the different impacts of various factors on regional carbon emissions. Select key policy measures to reduce carbon emissions based on regional characteristics. It is necessary to pay attention to the role of environmental regulation and implement environmental protection standards and environmental protection laws and regulations. It is also necessary to optimize the industrial structure and energy consumption structure according to the regional industrial characteristics and resource endowments, and promote the upgrading of industrial structure and green development. For example, for old industrial areas, such as the Northeast Economic Zone, adjusting the industrial structure and appropriately shifting the industrial center of gravity will help to rejuvenate new opportunities.
3. When formulating energy conservation and emission reduction policies, the synergistic emission reduction effect between regions should also be considered. It is necessary to make timely adjustments based on the contribution of carbon emission reduction driving factors in each economic zone and also to coordinate with China’s "*14th Five-Year Plan*" for a unified layout. At the same time, we must pay close attention to the promotion of "carbon trading" as a market-based emission reduction method to avoid fragmentation.

As mentioned in the full paper, while this paper innovatively considers the impact of environmental regulation on carbon emissions, it does not include all the influencing factors. In future studies, we can try to consider more influential factors, such as digital technology, digital ubiquity, etc. Financial benefits, etc., to obtain more comprehensive results (Supplementary Information).

## Supplementary Information

Supplementary Table S1.

## Data Availability

The datasets generated and analyzed during the current study are not publicly available, but are available from the corresponding author on reasonable request.
