# Supplementary material for: Research on spatial and temporal differences of carbon emissions and influencing factors in eight economic regions of China based on LMDI model
Source: Sci Rep. 2023 May 17;13:7965. doi: 10.1038/s41598-023-35181-w (PMC10192428; doi:10.1038/s41598-023-35181-w)
Supplement: Supplementary file 1 — Supplementary Table S1. [file 41598_2023_35181_MOESM1_ESM.docx]

**Appendix A**

**Table S1.** Decomposing the Cumulative Effects of Carbon Emissions Changes in China's Eight Economic Regions from 2008 to 2019

| **Area** | **Province** | **△C** | **△ECEI** | **△RIE** | **△RSE** | **△ISE** | **△EGE** | **△PSE** |
| --- | --- | --- | --- | --- | --- | --- | --- | --- |
| NEEZ | Liaoning | 11261.75 | -2932.68 | 36117.39 | -43009.55 | -16014.83 | 37650.81 | -549.40 |
|  | Jilin | 781.23 | 1254.29 | 3050.52 | -27235.02 | -1165.79 | 27847.83 | -2970.60 |
|  | Heilongjiang | 8328.00 | 3522.63 | 41590.09 | -33900.10 | -25388.42 | 28337.33 | -5833.52 |
| NEZ | Beijing | -4471.91 | -6055.74 | 22132.99 | -29067.42 | -2773.35 | 8823.53 | 2468.07 |
|  | Tianjin | 3691.73 | -3650.51 | 12050.72 | -15787.12 | -6724.19 | 14937.06 | 2865.77 |
|  | Hebei | 21233.65 | -2894.81 | -36781.99 | 5412.30 | -21947.36 | 72128.26 | 5317.25 |
|  | Shandong | 26513.37 | -2758.66 | 11402.36 | -44858.98 | -32751.83 | 88357.95 | 7122.52 |
| ECEZ | Shanghai | 4464.13 | 662.32 | -27754.88 | 17418.24 | -13300.29 | 23333.98 | 4104.76 |
|  | Jiangsu | 23174.19 | -3115.08 | -14002.99 | -26771.58 | -16243.38 | 76970.81 | 6336.39 |
|  | Zhejiang | 2695.51 | -11356.49 | -16236.84 | 669.03 | -8964.62 | 31358.17 | 7226.26 |
| SCEZ | Fujian | 8180.67 | -2785.55 | 13738.06 | -31362.72 | -973.16 | 26718.98 | 2845.06 |
|  | Guangdong | 13646.77 | -3672.74 | 23730.09 | -46669.60 | -11213.30 | 40429.81 | 11042.52 |
|  | Hainan | 1512.86 | -296.05 | 1887.55 | -2610.53 | -921.19 | 3025.00 | 428.07 |
| MYeREZ | Shaanxi | 25688.68 | 3597.82 | -6365.54 | -14484.41 | -5561.77 | 46058.95 | 2443.64 |
|  | Shanxi | 45182.95 | 22950.13 | 26685.14 | -46522.14 | -26938.08 | 67809.18 | 1198.72 |
|  | Henan | -2114.93 | -11812.59 | -26768.56 | -14985.04 | -14402.77 | 62922.72 | 2931.30 |
|  | Inner Mongolia | 65437.26 | 9516.84 | 48966.24 | -81151.70 | -4664.54 | 94328.86 | -1558.44 |
| MYtREZ | Hubei | 6737.41 | -3117.38 | 16667.64 | -51051.04 | -2399.36 | 45369.67 | 1267.88 |
|  | Hunan | 11652.08 | 1442.71 | 51955.62 | -89146.52 | -4904.05 | 50727.83 | 1576.49 |
|  | Anhui | 20664.33 | 2018.54 | -15786.76 | -11529.23 | -4997.87 | 50953.32 | 6.33 |
|  | Jiangxi | 35372.00 | 12212.26 | -38403.21 | 18159.72 | -8387.64 | 50829.33 | 961.54 |
| SWEZ | Yunnan | 823.25 | -12953.00 | 5089.11 | -25546.52 | -4855.83 | 38037.02 | 1052.48 |
|  | Guizhou | 9603.74 | -116.18 | 12662.37 | -41284.30 | -4003.59 | 40274.96 | 2070.48 |
|  | Sichuan | -1557.91 | -10386.59 | 19242.30 | -42261.94 | -5346.58 | 36503.56 | 691.35 |
|  | Chongqing | 3330.64 | -1544.50 | 19995.08 | -35290.47 | -1934.01 | 20242.49 | 1862.06 |
|  | Guangxi | 9558.91 | -1169.33 | 32752.36 | -42660.08 | -3295.74 | 23052.98 | 878.71 |
| NWEZ | Gansu | 4978.18 | -470.07 | 21058.61 | -25144.34 | -5736.11 | 15548.84 | -278.75 |
|  | Qinghai | 1983.56 | -973.28 | -2277.69 | -972.41 | -28.81 | 5908.42 | 327.33 |
|  | Ningxia | 20890.65 | -1210.59 | 30252.24 | -33167.70 | -3520.73 | 24850.00 | 3687.42 |
|  | Xinjiang | 37241.72 | 9631.65 | 16388.34 | -14858.58 | -10326.91 | 30158.55 | 6248.67 |
